# Integrating Distribution-Based and Anchor-Based Techniques to Identify Minimal Important Change for the Tinnitus Functional Index (TFI) Questionnaire

**DOI:** 10.3390/brainsci12060726

**Published:** 2022-05-31

**Authors:** Kathryn Fackrell, Deborah Ann Hall, Johanna Barry, Derek James Hoare

**Affiliations:** 1 NIHR Nottingham Biomedical Research Centre, Ropewalk House, 113 The Ropewalk, Nottingham NG1 5DU, UK; deborah.hall@hw.ac.uk (D.A.H.); derek.hoare@nottingham.ac.uk (D.J.H.); 2 Hearing Sciences, Mental Health and Clinical Neuroscience, School of Medicine, University of Nottingham, Nottingham NG7 2UH, UK; 3 School of Social Sciences, Heriot-Watt University Malaysia, No. 1, Jalan Venna P5/2, Precinct 5, Putrajaya 62200, Malaysia; 4 Department of Ear, Nose and Throat (ENT), Queen’s Medical Centre Campus, Nottingham University Hospitals (NHS) Trust, Derby Road, Nottingham NG7 2UH, UK; johanna.barry@touchime.org

**Keywords:** tinnitus-specific health-related quality of life, tinnitus diagnosis, outcome instruments, responsiveness, minimal important change

## Abstract

The Tinnitus Functional Index (TFI) was developed to be responsive to small treatment-related changes in the impact of tinnitus. Yet, no studies have integrated anchor-based and distribution-based techniques to produce a single Minimal Important Change (MIC) score. Here, we evaluated the responsiveness and interpretability of the TFI, determining for the first time a robust MIC score in a UK clinical population. Two-hundred and fifty-five patients with tinnitus participated in this prospective longitudinal validation study. Distribution-based estimates (Standard Error of Measurement, Smallest Detectable Change and Effect size) and anchor-based estimates of important change (minimal clinically important difference and Receiver Operator Curve optimal value) were calculated and then integrated using a visual anchor-based MIC distribution plot. A reduction in score of −14 was determined as the MIC estimate that exceeds the measurement error, most of the variability and reliably identifies patients demonstrating true improvement. It is therefore recommended that a reduction of 14 points should be used as a minimum change required when calculating statistical power and sample size in tinnitus intervention studies and assessing patients in clinical practice.

## 1. Introduction

The experience of tinnitus can involve much more than the sensation of sound. For many people it is a disorder that impacts on daily functioning, causes difficulties with sleep, listening and concentrating, impairs symptom-specific quality of life and results in poor psychological well-being [1]. There is a wide variety of treatments available to manage tinnitus symptoms including sound therapy, counselling and psychological therapies, neuromodulation, medications, and complementary and alternative therapies [2,3]. However, no treatment is universally effective, and evaluation of treatments in terms of what works for whom and in what circumstances is essential. To do this, clinicians and researchers rely on self-report questionnaires of tinnitus-specific health-related quality of life, of which a small number have been developed with known measurement properties for use as an outcome measure to assess treatment-related change over time (e.g., responsiveness) [4]. One questionnaire of this type is the Tinnitus Functional Index (TFI) [5]. First published in 2012, the TFI was developed to comprehensively measure a broad range of tinnitus-related complaints and was optimised to be responsive to change in tinnitus severity over time. To achieve this, several psychometric evaluations were undertaken, including estimates of floor and ceiling effects, effect sizes, and differences in ratings of mean change scores across global perception change categories (improved, no change, or worsened). All of these estimates were used to optimise the TFI’s ability to detect changes that truly reflect improvements in tinnitus impact [5,6].

Following the development of the TFI, Meikle et al. [5] proposed a reduction in TFI scores of 13 points as a preliminary minimal clinically meaningful score, referred to as minimal important change (MIC), based on their validation using anchor-based techniques (external indictors that define change scores based on patient perspective). Since then, a range of alternative scores (change of 4.8 to 22 points) have been proposed as representing the minimal change in scores using either anchor-based [7] or distribution-based techniques (statistical properties of the sample) [8,9,10,11]. Our previous work [8,12] evaluating the structure of the TFI also recommended that the auditory items (items 13, 14, 15) should not be included in the calculation for the global TFI score as including these items risks unduly diluting the specificity and sensitivity of the questionnaire. We referred to this shorter version as TFI-22. The impact on the responsiveness from not including these items as yet has not been evaluated. In the field of psychometrics, there has been a clear move towards integrating multiple estimates from anchor-based and distribution-based techniques to identify a single MIC or a narrow range of values that have both external validity and account for variability [13,14,15]. This can be achieved by using a visual anchor-based MIC distribution plot to examine score distributions and compare estimates [15]. No tinnitus study has previously integrated anchor-based and distribution-based techniques to identify a single MIC score or range of scores. In the present study, we examined the responsiveness and interpretability of the TFI (including TFI-22) in a large clinical sample of UK NHS patients treated for tinnitus, with the objective of integrating anchor-based and distribution-based estimates to determine an MIC and narrow range of scores for the TFI that are clinically meaningful, account for patient perceived benefits, and provide an assessment of measurement error.

## 2. Materials and Methods

This was a prospective multi-site, repeated-measures validation study. Ethical approval was granted by the Cornwall and Plymouth Research Ethics Committee (13/SW/0234) and the Nottingham University Hospitals National Health Service (NHS) Trust was Sponsor. For an extended description of eligibility and data collection schedule see Fackrell et al. [12].

### 2.1. Data Collection Schedule

Participants completed the TFI in clinic before or immediately after their initial NHS audiology appointment for diagnostic assessment (T0), and then at home at 3 months (T1), 6 months (T2), and 9 months (T3) after T0. All participants that completed the study were entered into a prize draw to win one of three GBP 50 gift vouchers.

### 2.2. Inclusion Criteria

The inclusion criteria were (i) adult patients (≥18 years old) reporting persistent tinnitus attending their first appointment with an NHS audiologist, and (ii) sufficient standard of written English to independently complete the study questionnaires.

### 2.3. Participants

Detailed participant characteristics are reported in Fackrell et al. [12]. A total of 255 tinnitus patients (male: 149; female: 105; 1 prefer not to say) from 12 NHS audiology clinics completed the TFI at T0, 198 (78%) completed follow-up questionnaires at T1, 176 (69%) at T2, and 166 (65%) at T3. Compliance exceeded the anticipated rate of 62% (based on Vernon et al., findings [16]) possibly due to our use of personalised reminders. At T1, 139 (55%) patients reported having tried a variety of tinnitus treatments. Fewer patients reported treatments at T2 (*n* = 31) and T3 (*n* = 22). The most commonly reported treatments were hearing aids and portable sound generating devices. Other reported treatment included tinnitus maskers, medications for sleep, relaxation training, yoga, mindfulness, and hypnosis.

### 2.4. Measures

#### 2.4.1. Tinnitus Functional Index

The TFI measures the functional impact of tinnitus using 25 items, each rated on an 11-point Likert scale with descriptors at either end of the scale [5]. Patients rate each item according to how they have felt over the past week. To calculate the TFI global score, the sum of all item scores is divided by 2.5 to give a score out of 100. The TFI encompasses eight subscales: (i) Intrusiveness, (ii) Sense of control, (iii) Cognition, (iv) Sleep, (v) Auditory, (vi) Relaxation, (vii) Quality of life, and (viii) Emotional distress. Our previous work [8,12] recommended to not include the auditory items (items 13, 14, 15) in the global score calculation, therefore dividing the sum of the remaining 22 items by 2.2 to give a global score out of 100. This shorter version is referred to as the TFI-22 throughout, while TFI refers to the 25-item questionnaire. Higher scores indicate a greater impact on everyday functioning. The TFI and TFI-22 global scores are classified based on TFI grading system for the UK (no problem; small; moderate; big; very big problem) [12]. Scores from T0, T1, T2, and T3 were considered in the analyses reported here.

#### 2.4.2. Global Rating of Perceived Problem with Tinnitus (Perceived Problem Rating)

At T0 patients completed a single question: “How much of a problem is your tinnitus?”, with five possible response options: 1 = not a problem, 2 = a small problem, 3 = a moderate problem, 4 = a big problem, and 5 = a very big problem.

#### 2.4.3. Clinical Global Impression of Perceived Change in Tinnitus (CGI)

At each follow-up assessment, patients answered one question about the extent to which their tinnitus changed: “All things considered, how is your overall tinnitus condition now, compared to x months ago?”, where x = 3, 6, and 9 months at T1, T2, and T3, respectively. Responses were made on a 7-point scale (3 = ‘much improved’, 2 = ‘moderately improved’, 1 = ‘slightly improved’, 0 = ‘no change’, −1 = ‘slightly worse’, −2 = ‘moderately worse’ to −3 = ‘much worse’). Responses from T1, T2, and T3 were considered in analyses.

### 2.5. Analysis Methods

Distribution-based and anchor-based techniques to evaluate responsiveness and interpretability were performed in SPSS v.21.0 [17] and Microsoft Excel. The TFI subscales have been proposed as having use as standalone measures [5] so where possible were subject to analyses. A criterion group approach was used for all analyses, in which the TFI global and subscale scores were stratified according to CGI scores [18]. CGI categories were used to assign patients into distinct categories that had qualitative meaning related to patient experience [14,19,20]. To explore the adequateness of the CGI as an anchor, correlation coefficients (Spearman’s Rho) were examined between CGI categories and the TFI scores, with correlation <0.4 taken as indicating adequateness. CGI response categories were collapsed from the 7-point scale to a 3-point scale of ‘improved’ (much to slightly improved), ‘no change’, and ‘worsened’ (slightly to much worse) to ensure sufficient sample sizes for some of the analysis described below.

#### 2.5.1. Visual Anchor-Based Minimal Important Change (MIC) Distribution

To identify an MIC score or a narrow range of values that have both external validity and accounts for the variability, we performed the recommended visual examination of the score distributions and triangulated distribution-based and anchor-based estimates [15,20,21]. Estimates were depicted using the visual anchor-based MIC distribution plot [15], whereby proportional frequencies of the TFI change scores for the ‘improved’ and ‘no change’ CGI (3-point) categories were plotted as distribution curves (independent of sample size), with all change estimates plotted with the distributions. The preferred MIC value should desirably account for both patient experience and measurement error, but priority should be placed on the patient experience [15,22]. Most data were available for T1–T0, therefore the global TFI change scores for T1 were used in the visual anchor-based MIC distribution plot. The sample size for patients selecting ‘worsening’ of tinnitus on the CGI was too small (*n* = 26 at T1) to confidently interpret the results, therefore the proportional frequencies were not plotted for ‘worsened’ categories.

#### 2.5.2. Distribution-Based Techniques

The recommended distribution-based techniques Standard Error of Measurement (SEM) and Smallest Detectable Change (SDC) were used to identify measurement error, whilst Effect Size (ES) was used to identify the magnitude of change [23,24].

The SEM provides information on the precision of the TFI measurement and was calculated as SD*diff*/√2. To account for the total shared variance over the time intervals, a one-way ANOVA was conducted for each analysis to identify the SD*diff* [25]. SEM is expressed in the same units of measurement as the TFI scores and was therefore reasonably easy to interpret, with larger SEM scores being equivalent to lower reliability [22,26,27]. It has been proposed that one SEM may be equivalent to an MIC score [20,28]. Alternatively, the SEM estimate multiplied by four has also been suggested as additionally accounting for the variability in individual scores over time and both Type I and Type II errors [21]. Both methods were considered in the visual anchor-based MIC distribution plot to identify an MIC score.

SDC reflects the extent of expected measurement error and was derived from the SEM between repeated measures [22,29]. It was calculated as 1.96 × √2 × SEM. To calculate the SEM and SDC, responses from a subset of patients that reported ‘no change’ on the CGI at T1 and T2 were used. The sample size for the ‘no change’ subgroup at T3 was too small (*n* = 29) for analysis. Patients who identified themselves as having ‘no change’ on the CGI, but had changes in TFI scores of >70 were considered outliers (large change scores such as this would correspond to a change from severe tinnitus to mild tinnitus or vice versa (see [12] for grading system). TFI data from one patient were removed as outliers (global and subscales). Therefore, with no other missing data, the effective sample size was 50. Subscale data for four other patients (not including the patient mentioned above) were removed as outliers.

ES provides information on the magnitude of the score and does not account for any error in measurement [22]. The 3-point CGI categories (‘improved’, ‘no change’, and ‘worsened’) were used to calculate ES for T1–T3 TFI and TFI-22 data. ES was calculated as:

$$\mathrm{ES}=\frac{x_{1}-x_{0}}{\sqrt{\frac{\sum {\left(x_{0}-{\bar{x}}_{0}\right)}^{2}\;}{n-1}}}$$

where *x*_0_ refers to pre-test score and *x*_1_ is the post-test score, divided by the SD of the pre-test scores [19]. The standard criteria for the ES estimates are: ≥0.20 is a small effect, 0.5 is a medium effect, and ≥0.80 is a large effect [30,31]. It is expected that ES estimates would be large positive values in patients reporting improvements, small–medium negative values in patients reporting worsening and small values (close to zero) in patients reporting ‘no change’. It has been proposed that ES estimates of 0.5 (or ½ SD) may approximate the minimum value needed to show a clinically meaningful change [32,33]. Therefore, the ½ SD (ES = 0.5) was calculated for the improved group using the baseline SD and was considered in the visual anchor-based MIC distribution plots.

#### 2.5.3. Anchor-Based Techniques

The anchor-based techniques evaluating mean change and Receiver Operator Characteristics (ROC) were used to assess meaningful change in scores [33,34,35]. Because the degree of severity at baseline can influence the minimal perceived change [13,36,37], change scores compared to baseline values for improvement were evaluated.

The mean change in TFI, TFI-22 global and subscale scores between baseline (T0) and follow-up (T1–T3) were calculated for each CGI category. The difference (referred to as minimal clinically important difference, MCID) between the mean change scores for ‘no change’ and ‘slightly’, ‘moderately’, and ‘much’ improved/worsened groups for T1–T3 were calculated and tabulated. The MCID scores for ‘slightly’ and ‘moderately’ improved are considered in the visual anchor-based MIC distribution plots. To examine the effects of baseline values, the mean change in global TFI and TFI-22 scores between baseline (T0) and follow-up (T1–T3) were calculated corresponding to each of the 3-point CGI categories (‘improved’, ‘no change’, and ‘worsened’) but stratified within the grading system (from small problem to very big problem).

ROC analysis was used to detect the threshold value that best discriminates between the patients who improved or worsened from those who perceived no change [38,39]. Sensitivity was equivalent to the probability that the proportion of improved (or worsened) patients were correctly classified according to their TFI score as improving (or worsening), whilst specificity was defined by the probability that patients were correctly classified as experiencing “no change” in tinnitus. An Area Under the ROC Curve (AUC) value of <0.7 was taken to indicate the ability of the TFI to successfully discriminate change [38,40]. A balance between sensitivity and specificity was employed to identify the optimal threshold value for detecting a meaningful change. To evaluate the sensitivity of the global TFI and TFI-22 to correctly classify improvements, ROC curves were calculated for each comparison between ‘no change’ and ’slightly’ and ‘moderately’ improved groups. To evaluate the sensitivity of the TFI subscales to correctly classify improvements, and of the TFI and TFI-22 to correctly classify worsening, ROC curves were calculated using 3-point CGI categories (‘improved’, ‘no change’, and ‘worsened’).

## 3. Results

### 3.1. Descriptive Statistics

The mean TFI, TFI-22 global and subscales scores from T0 to T3, alongside the mean TFI and TFI-22 scores and distribution of patients within the grading systems are presented in Supplementary Table S1. According the TFI grading system (UK), tinnitus was a moderate to big problem for most patients at T0, with very little variability across the subsequence time intervals. The mean change scores on the TFI, TFI-22 and subscales categorised according to the responses to the CGI are presented in Table 1. Very few patients reported worsening of their tinnitus in the first 6 months, although the number reporting worsening did increase by 9 months. The sample sizes for the ‘much improved’ and ‘much worse’ categories were not sufficient to make meaningful comparisons so were only used as information about the population (Table 1). The mean TFI, TFI-22 and subscales change scores within the CGI categories collapsed into ‘improved’, ‘no change’, and ‘worsened’ are presented in Supplementary Table S2.

Spearman’s Rho indicated moderate correlations between the global TFI and TFI-22 scores and the CGI at T1, T2, and T3 (Spearman’s Rho = 0.4–0.5) (Supplementary Table S3). The correlations for the subscales, on the other hand, ranged from moderate to weak (Spearman’s Rho = 0.2–0.6), suggesting that the TFI subscales scores may not be reflecting the ratings of change. Therefore, the CGI ratings may not be the most appropriate anchor for the subscales and any analysis should be interpreted with caution.

### 3.2. Distribution-Based Estimates

The SEM and SDC for global TFI, TFI-22 and subscale scores comparing T0 data and pooled T1 and T2 data are presented in Table 2. The SEM estimate for the global TFI and TFI-22 scores were comparable, both scores indicating minimal measurement error (5.1 and 5.0, respectively, out of a possible 100). However, this led to large estimates when the SEM was multiplied by four. The SDC scores for the global TFI and TFI-22 scores were again comparable with a slightly smaller estimate for TFI-22 (13.9) than the TFI (14.2). These estimates are considerably smaller than the SEM × 4 estimates. In terms of the TFI subscales, the SEM and SDC estimates were in general considerably larger than the estimates observed for the global score, and there were large inconsistencies between the estimates for SDC and SEM × 4 (Table 2), all of which indicates potentially large measurement error associated with the subscales.

**Figure 1:**
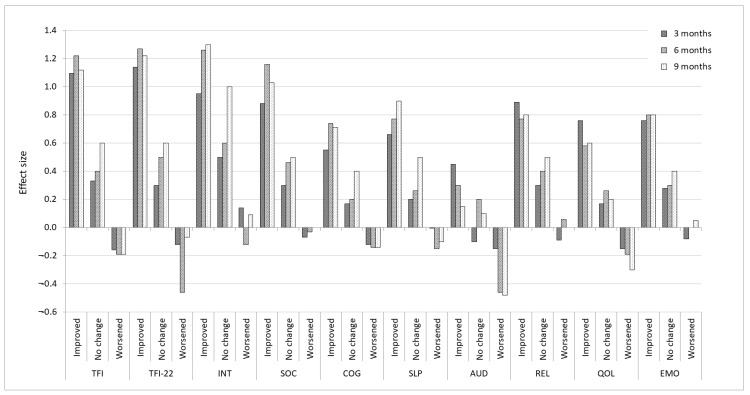
Effect sizes for TFI, TFI-22 global and subscales corresponding to three Clinical Global Impression categories (‘improved’, ‘no change’, ‘worsened’) at follow-up administrations. ES estimates are: ≥0.20 is a small effect, 0.5 is a medium effect, and ≥0.80 is a large effect [29].

For the ‘improved’ groups, large ES were observed for the global TFI and TFI-22, ranging from 1.1 to 1.2, for all time intervals as expected (Figure 1). The estimated baseline SD for the improved group was 19.8, resulting in a minimal score of 9.9 (based on ES of 0.5). As expected, small negative ES were observed for the TFI and TFI-22 ‘worsened’ groups (ranging from 0.1 to 0.4). The ES for the ‘no change’ groups were considerably larger than expected but was comparable to those reported by Meikle et al. [5]. A similar pattern was observed for the subscales, except for the Auditory subscale for which a small ES was observed for the ‘improved’ groups and moderate ES for ‘worsened’ groups.

### 3.3. Anchor-Based Estimates

#### 3.3.1. Identifying Meaningful Change for Improvement

Mean change scores on the TFI, TFI-22, and subscales according to CGI categories showed a pattern with increasing scores from ‘much improved’ to ‘much worse’ for the time intervals, except ‘moderately worse’ at T1 presumably due to a small sample size (Table 1).

The MCID between the ‘no change’ and ‘slightly improved’ groups at T1 indicated that a minimum decrease in TFI and TFI-22 scores of −8.8 and −8.5, respectively, should reflect meaningful improvements in tinnitus for patients (Table 1). However, the MCID between these two groups decreased over time, suggesting that smaller changes become more important at later time points. In contrast, the MCID between ‘moderately improved’ and ‘no change’ was more consistent over time, suggesting that a reduction of −14 in scores would indicate meaningful improvements in tinnitus for patients.

MCIDs stratified by baseline grading group (‘small problem’ to ‘very big problem’) showed that the degree of change between the ‘improved’ and ‘no change’ categories differed depending on the baseline value across all time intervals. Patients with higher baseline scores were more likely to report larger changes in scores to register an improvement than those reporting fewer problems at baseline. For example, MCID at T1 ranged from −5.5 for patients reporting a small problem with their tinnitus at T0 to −13.9 for patients reporting a big problem with their tinnitus at T0 (Table 3).

**Figure 2:**
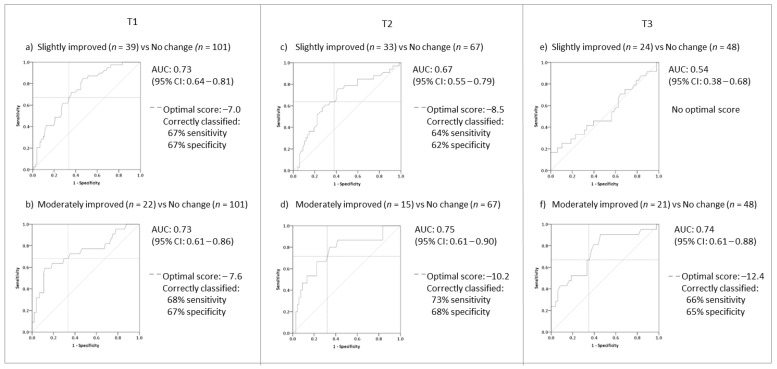
Receiver operating characteristic (ROC) curves with optimal values for identifying improvements above no change using the TFI global scores. (**a**,**c**,**e**) = patients who reported ‘slight improvements’ with those who reported ‘no change’ in their tinnitus for T1 (3 months), T2 (6 months), and T3 (9 months); (**b**,**d**,**f**) = patients who reported ‘moderate improvements’ with those who reported ‘no change’ in their tinnitus for T1 (3 months), T2 (6 months), and T3 (9 months); solid light grey line indicates 50% probability of correctly classifying improvement. AUC = Area Under the Curve.

ROC analyses were conducted for the TFI and TFI-22 global scores comparing patients who reported ‘slight’ (*n* = 39) and ‘moderate’ (*n* = 22) improvements on the CGI with those who reported ‘no change’ (*n* = 101) in their tinnitus at T1–T3 (Figure 2; Supplementary Figure S1). AUC values for ‘slightly improved’ versus ‘no change’ only exceeded the recommended criteria at T1 (AUC < 0.7), with the optimal cut-off score between sensitivity and specificity identified as −7.0 points on both versions (Figure 2a). This suggests that there is a good level of accuracy at identifying improvement based on small changes after 3 months, but this accuracy reduces at 9 months from baseline (AUC = 0.5). In contrast, the AUC values observed for ‘moderately improved’ vs. ‘no change’ exceeded the criteria across all time intervals (AUC < 0.7). The optimal cut-off score for the TFI at T1 was close to that reported for ‘slightly improved’ (−7.6), whilst the cut-off score at T2 and T3 gradually increased to −12.4 (Figure 2). The TFI-22 showed a similar pattern (Supplementary Figure S1). Given the consistency of data at T1 and similarity in scores, the optimal score identified for T1 data (−7.6) is considered in the visual anchor-based MIC distribution plots.

The mean change scores for the TFI subscales according to the CGI categories followed the same pattern as the global TFI and TFI-22, however the MCID between ‘slightly improved’ and ‘no change’ indicated large variability between subscales, ranging from −2.3 (Cognition) to −12.1 (Auditory) at T1 (Table 1). These differences were not consistent over time for any subscale. The AUC values for subscales comparing patients reporting ‘improvements’ with patients reporting ‘no changes’ were all below the recommended criteria for the time intervals (Table 4). Possible exceptions were AUC values at T1 for the Intrusiveness, Sense of Control, Relaxation and QoL subscales, which were only marginally below the criteria at 0.69, suggesting that these subscales could accurately detect improvements. There was variability in the optimal cut-off scores for these subscales, with scores ranging from −6.3 (QoL) to −13.3 (relaxation) (Table 4).

#### 3.3.2. Identifying Meaningful Change for Worsening

The MCIDs between the ‘no change’ and ‘slightly worse’ subgroups were reasonably consistent over time, ranging from +6.5 to +8.5 (Table 1). A minimum increase in TFI and TFI-22 scores of 8 would indicate slight worsening that is meaningful to patients. The sample size for ‘moderately worse’ at T1 and T2 was insufficient for comparisons at these time intervals (Table 1). At T3, the MCID between ‘no change’ and ‘moderately worse’ suggested that an increase in TFI and TFI-22 scores of 16 indicates moderate worsening of tinnitus.

ROC analysis was conducted comparing patients reporting ‘worsening’ of their tinnitus with those reporting ‘no change’ (Table 4). Whilst AUC values at T1 and T2 for the TFI and TFI-22 were below the recommended criteria, at T3 the AUC value of 0.7 did meet the criteria (Table 4). The optimal cut-off scores varied across time intervals, and slightly differed across the two versions, with higher cut-off values for the TFI-22 (range: 1.6 to 4.1) than the TFI (range: 1.4–2.8). The difference between the AUC values and cut-off scores could be attributed to more patients reporting their tinnitus had worsened at T3 adding more stability to the comparisons. Therefore, the global TFI and TFI-22 could discriminate patients whose tinnitus had become worse from those whose tinnitus did not change, with optimal cut-off score of 2.8 and 4.1, respectively. Again, subscales varied in the magnitude of change between ‘worse’ and ‘no change’ (Table 1). Due to the variability observed in the subscale data for detecting ‘improvements’ and the lower AUC values for the global TFI and TFI-22 scores for detecting ‘worsening’, the TFI subscales were not subjected to ROC analysis for worsening.

### 3.4. Visual Anchor-Based MIC Distribution

**Figure 3:**
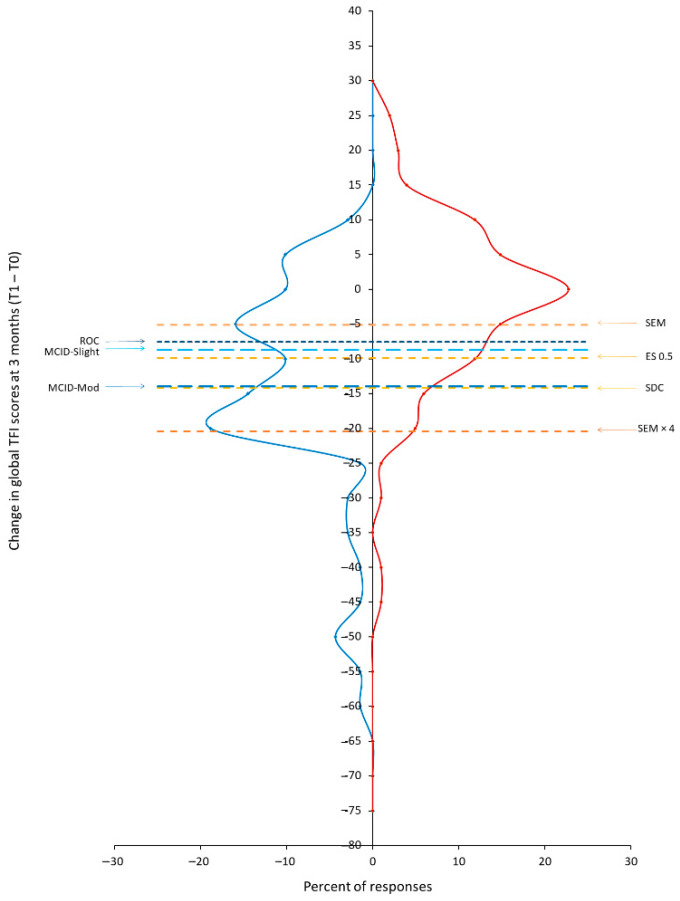
Distributions (expressed in percentages) of the changes in TFI global scores for tinnitus patients who reported improvements in tinnitus (blue distribution line) and those who reported no change in tinnitus at 3 months from baseline (red distribution line). SEM = Standard Error of Measurement, SEM × 4 = Standard Error of Measurement multiplied by 4, ROC = Receiver Operating Characteristic (ROC) optimal value, SDC = Smallest Detectable Change, MCID-Slight = Minimal Clinically Important Difference estimates for ‘slightly improved’, MCID-Mod = Minimal Clinically Important Difference estimates for ‘moderately improved’.

The proportional frequencies of the global TFI and TFI-22 change scores according to patients reporting ‘improvements’ and ‘no change’ at T1 were plotted in visual anchor-based MIC distributions. SEM, SDC, and ES 0.5 estimates, MCIDs for ‘slightly’ and ‘moderately’ improved and the optimal ROC value were also plotted (Figure 3; Supplementary Figure S2).

For both the TFI and TFI-22, the SEM was the lowest estimate (5.1 and 5.0, respectively), suggesting variability in the precision of measurement was reasonably small. However, it was not equivalent to the MCID estimates and was clearly within a large amount of variability between the two distributions. The ES 0.5 estimate (−9.9) for ‘improved’ group, the MCID for ‘slightly improved’ (−8.8) and the ROC optimal value for ‘slightly improved’ (−7.6) were slightly above the SEM estimate. Inspection of the two distributions at this point suggests that a reasonable proportion of patients reporting ‘no change’ group would still be identified; there was still reasonably high variability in the data beyond the estimates, which may inflate change scores. The SEM × 4 suggested that a decrease of >20 points was required to be above the variability and although the proportion of patients reporting ‘improvements’ peaked at this point, it considerably reduced after, whilst the variability in ‘no change’ was only marginally reduced. By contrast, the MCID for ‘moderately’ improved (−14), was equivalent to the SDC estimate (−14). This was associated with fewer patients reporting ‘no change’ (so there is smaller variability) and a peak in the number of patients identifying ‘improvement’ after this point; this indication overcomes the majority of the variability, exceeds the measurement error and would clearly identify patients with true improvement. As such, we have determined the MIC for the TFI and TFI-22 as a reduction in scores of −14.

## 4. Discussion

The current study involved a comprehensive psychometric evaluation to determine an MIC score for the TFI that takes account of both patient perceived benefit and measurement error. We conclude for a UK clinical population that a reduction in global TFI or TFI-22 scores of 14 points indicate a change that is meaningful for patients and above measurement error. This value should be used to interpret individual patient progress in clinical practice, and as a minimum change required when calculating statistical power and sample size in a tinnitus intervention study [20,29].

Maximising responsiveness to change was a key factor in the development of the TFI. Items were specifically chosen because they describe attributes that are likely to undergo changes following intervention and an MIC was calculated using an anchor-based technique [5]. Meikle et al. [5] concluded that the TFI was a responsive measure of change, showing large ES for patients who perceived improvements. Importantly, Meikle et al. [5] suggested that a reduction in TFI global score of approximately 13 points as an interim indicator of a meaningful improvement. The large ES and the ability to effectively measure change in tinnitus impact over time were also observed in this study and a similar reduction in global TFI scores was identified (−14 points) as an MIC.

However, the sensitivity of the TFI to change has been debated in the literature and a number of alternative interpretations have been proposed [6,7,8,9,10,11,41,42,43]. Firstly, our previous work [8] evaluating the TFI in a research participant (non-clinical) population suggested that a reduction in TFI scores of ≈22 points was needed for a “true change” above measurement error. This was considerably larger than preliminary score by Meikle et al. [5] and identified here. One possible explanation for this disparity was that patient perception of change (CGI) was not measured in our previous work and therefore the large variability in scores observed between test–retest may have reflected an assumption that the impact of tinnitus would remain stable over the 2-week period. Tinnitus patients can often experience changes in their tinnitus and adjust their perception of the impact, because of new stressful event occurring, or through natural coping mechanisms, or re-evaluation of internal standards of health-status [44,45,46]. Consequently, the natural variability of tinnitus over the days and weeks could have inflated the observed measurement error and SDC estimates in this population. In contrast, Chandra et al. [11] identified a lower SDC estimate of 4.8 for the TFI in a New Zealand research (non-clinical) population so did not observe the same variability across the 2-week period. This score is considerably smaller than the MIC (and SDC) score identified here. One possible explanation is that variations in responses are often observed across different populations. Culture, values, language, and other psychosocial characteristics have been shown to affect tinnitus perception and as such may have reduced the variability in tinnitus perception across the 2-week period [47]. However, Chandra et al. [11] did not account for patient perception of change and as such do not know if a change in TFI scores of 4.8 would be meaningful to patients. Alternatively, Folmer [9] argued that a reduction in scores of about 7 points would indicate significant change. However, this score was based on the statistical significance of the scores and ES calculations. The magnitude of ES does not indicate the precision of measurement or determine the validity of the change score [48,49]. In other words, it would be near impossible to identify whether the magnitude of effect reflects the intervention or the error in measurement. Furthermore, in the current study, we observed small to medium effects for the TFI global and subscale scores in ‘no change’ groups. As researchers, we need to be conscious of this natural variability when making judgements on the significance of treatment effects and when claiming that a scale is responsive to change.

Most recently, Skarżyński et al. [7] used the CGI anchors (‘no change’ compared to ‘much to very much improved) following stapedotomy surgery to determine an MIC score of −8.8 points in TFI scores as meaningful change. This MIC score is again somewhat different from those identified here. It is possible that perception of improvement could depend on the intervention or treatment being received. Our study included patients who were receiving a broad range of treatments or no treatments at all in order to identify an MIC that could be used in every clinical or research situation not just specific treatments. It is also possible that the MIC score proposed by Skarżyński et al. [7] was lower than expected because it did not account for the measurement error or precision. Future research should investigate whether patient perceptions of improvements do differ across treatment-specific populations and adjust for measurement error when calculating MIC scores.

Importantly, in this study, the MIC score was identified by integrating anchor-based estimates based on patients’ ratings of perceived change and ROC optimal values with distribution-based estimates based on the statistical properties of the scores and measurement error. By using a visual anchor-based MIC distribution plot, we were able to visually examine the score distributions alongside these estimates and identified a reduction in global TFI/TFI-22 scores of −14 points as an MIC. Although this MIC score is similar to the preliminary score proposed by Meikle et al. [5], it accounts for meaningful improvement above measurement error and the majority of variability in scores, and overcomes to some degree the variable nature of patient perceived meaningful change. In other words, the −13 points proposed by Meikle et al. [5] would be slightly more susceptible to identifying treatment-related changes that may not reflect meaningful change for all those patients. Consequently, given the main purpose of the TFI is to be a sensitive measure to small but important treatment-related changes, the MIC recommended here is a more reliable estimate than those calculated previously, and we can be confident that the change identified is a realistic reflection of true change in score. Furthermore, not including the Auditory items in the calculation for the global TFI score, as previously recommended [8,12], had no detrimental impact on the responsiveness of the TFI. The results clearly demonstrated that there was no increase in measurement error or decrease in the accuracy to identify change due to removing these items from the global score. Therefore, the calculation for TFI-22 can be confidently used when assessing treatment-related changes.

In the current study, the MCID scores corresponding to each CGI category were dependent on the baseline grading. Patients with higher baseline TFI global scores required bigger changes in scores than patients with lower baseline scores to register an important change. A similar pattern in minimal change corresponding to baseline values was also observed for the TQ [50]. Logically, patients with high scores at baseline have more opportunity to register improvements than those with low scores at baseline. In our study, the MIC score (−14) identified without consideration of baseline values was still larger than the MCID estimates associated with the ‘big to very problems’ at baseline and therefore would account for these differences. Researchers and clinicians should therefore be mindful of baseline values and the MCID scores reported here when evaluating the effectiveness of treatment in patients experiencing different degrees of tinnitus impact. For example, patients with high baseline scores are less likely to notice smaller changes and may require a larger change for it be meaningful. Future studies should examine the measurement error associated with the different baseline values and whether this variability is seen in other patient or research populations.

The magnitude of change is dependent on the timeframe in which the scores are compared. The ability of the TFI to detect improvements did not extend past 6 months from baseline. At 9 months, difference scores and the magnitude of change for improvements was notably lower than previous time points, and consequently, patients experiencing improvements were difficult to discriminate from those who remained unchanged. Therefore, consideration should be placed on the time-intervals when collecting follow-up data in clinical practice or planning clinical trials as the magnitude of change would vary depending on the time-intervals selected. In contrast, the ability of the TFI to detect worsening of tinnitus was greatest at 9 months, possibly because there were more patients reporting worsening at 9 months than at earlier time points, hence more power. This could also reflect the patient population, and the natural history of tinnitus. Whilst there is evidence that tinnitus generally improves over time without intervention [51], a recent longitudinal study found that 9% of participants reported tinnitus was worse 4 years after its onset [46], although it was not known whether participants received any clinical help for tinnitus during this time.

One limitation with the study was that we were unable to fully assess the responsiveness and interpretability of the TFI subscales. The anchor measure used (CGI) may not accurately reflect changes associated with the concept each subscale is measuring. Therefore, until further assessment has been conducted, it is recommended that subscales should be used with caution when interpreting treatment-related change. Another limitation was that due to the small sample of patients whose tinnitus became worse at 3 or 6 months, we were unable to determine an MIC for worsening at these time points. However, we did observe that the TFI discriminated patients whose tinnitus had become worse from those whose tinnitus did not change.

## 5. Conclusions

This is the first report to integrate both anchor-based and distribution-based techniques and that accounts for both patient perceived benefit and measurement error, thereby identifying a minimal important change score of −14 points for the TFI and TFI-22. Additionally, given that MIC estimates are clearly dependent on the population and baseline scores, it is recommended that researchers incorporate the CGI of perceived change question into all clinical trials; this would provide additional support for the MIC and could be used to identify the degree of variability in participants who perceived ‘no change’ in their tinnitus following an intervention or across time intervals. Although ES estimates can be used as evidence of identifying change if the direction and magnitude follow the expected pattern, they should not be used as standalone evidence of change. The responsiveness and accuracy of TFI-22 was confirmed to be similar to the TFI, with the same MIC recommended for both calculations. Clinicians and researchers can therefore feel confident using the TFI-22 to measure outcome. This study provides further evidence that the TFI is a responsive measure to change and should be used in clinical trials of tinnitus treatments.

## Figures and Tables

**Table 1:** Mean (SD) TFI, TFI-22 global and subscale scores according to the Clinical Global Impression (CGI) categories and Minimal Clinically Important Difference (MCID) for ‘improved’ and ‘worsened’ categories for each subscale at follow-up administrations.

		T1			T2			T3		
	CGI	*n*	Mean (SD)	MCID	*n*	Mean (SD)	MCID	*n*	Mean (SD)	MCID
TFI	Much improved	8	−37.2 (22.9)	−33.1	19	−29.4 (16.5)	−22.3	14	−29.1 (19.9)	−20.0
	Moderately improved	22	−16.6 (16.5)	−12.6	15	−21.7 (17.7)	−14.6	21	−22.8 (18.5)	−13.7
	Slightly improved	39	−12.8 (10.2)	−8.8	33	−12.7 (11.8)	−5.6	24	−12.0 (16.7)	−2.9
	No change	101	−4.1 (12.0)		67	−7.1 (13.5)		48	−9.1 (12.7)	
	Slightly worse	23	2.5 (12.1)	6.5	30	1.4 (14.9)	8.5	33	−0.7 (16.1)	8.4
	Moderately worse	3	−2.5 (11.4)	1.5	9	6.0 (13.4)	13.2	14	7.2 (20.5)	16.3
	Much worse				2	12.2 (22.3)	19.3	11	8.1 (10.3)	17.2
TFI-22	Much improved	8	−37.7 (23.3)	−32.7	19	−31.4 (17.4)	−23.8	14	−31.9 (19.2)	−21.9
	Moderately improved	22	−18.4 (18.1)	−13.5	15	−21.4 (17.0)	−13.8	21	−24.7 (18.3)	−14.6
	Slightly improved	39	−13.5 (10.9)	−8.5	33	−14.7 (12.4)	−7.1	24	−14.7 (17.0)	−4.7
	No change	101	−4.9 (12.0)		67	−7.6 (14.5)		48	−10.0 (13.6)	
	Slightly worse	23	2.4 (14.0)	7.4	30	0.4 (15.4)	8.0	33	−2.1 (17.0)	7.9
	Moderately worse	3	−3.6 (11.8)	1.3	9	4.2 (13.5)	11.9	14	5.8 (21.6)	15.8
	Much worse				2	11.8 (22.5)	19.4	11	6.3 (10.7)	16.3
INT	Much improved	8	−54.6 (24.2)	−47.6	19	−36.5 (24.2)	−24.6	14	−35.7 (19.8)	−17.5
	Moderately improved	22	−16.4 (18.0)	−9.4	15	−25.3 (25.0)	−13.4	21	−30.3 (14.8)	−12.1
	Slightly improved	39	−15.1 (16.5)	−8.1	33	19.7 (18.1)	−7.8	24	−21.1 (23.3)	−2.9
	No change	101	−7.0 (22.4)		67	−11.9 (18.1)		48	−18.2 (22.6)	
	Slightly worse	23	−4.2 (23.8)	2.8	30	3.9 (23.5)	15.8	33	−8.4 (22.5)	9.8
	Moderately worse	3	−11.1 (15.4)	−4.1	9	2.6 (16.1)	14.5	14	3.1 (19.5)	21.3
	Much worse				2	5.0 (7.1)	16.9	11	7.9 (26.0)	26.1
SOC	Much improved	8	−50.8 (27.2)	−46.7	19	−40.5 (21.8)	−30.8	14	−34.5 (30.0)	−24.4
	Moderately improved	22	−19.4 (28.6)	−15.2	15	−21.6 (26.9)	−11.8	21	−24.6 (23.6)	−14.5
	Slightly improved	39	−15.3 (17.7)	−11.1	33	−19.4 (16.0)	−9.6	24	−15.1 (22.4)	−5.0
	No change	101	−4.2 (18.3)		67	−9.8 (23.7)		48	−10.1 (20.2)	
	Slightly worse	23	−0.6 (14.1)	3.6	30	−0.9 (26.0)	8.9	33	−4.6 (18.7)	5.5
	Moderately worse	3	36.7 (49.8)	40.8	9	3.7 (12.6)	13.5	14	4.3 (33.0)	14.4
	Much worse				2	11.7 (16.5)	21.4	11	5.5 (10.1)	15.6
COG	Much improved	8	−32.5 (25.0)	−27.7	19	−24.9 (22.6)	−20.1	14	−23.8 (28.4)	−17.1
	Moderately improved	22	−13.9 (21.9)	−9.1	15	−13.9 (20.4)	−8.7	21	−20.6 (26.7)	−13.9
	Slightly improved	39	−7.1 (18.8)	−2.3	33	−11.0 (17.8)	−6.2	24	−16.9 (25.8)	−10.2
	No change	101	−4.8 (21.6)		67	−4.8 (19.5)		48	−6.7 (15.4)	
	Slightly worse	23	3.5 (20.7)	8.3	30	0.8 (18.9)	5.6	33	1.4 (22.0)	8.1
	Moderately worse	3	−2.2 (19.0)	2.6	9	5.9 (17.9)	10.7	14	7.6 (32.6)	14.4
	Much worse				2	20.0 (47.1)	24.8	11	4.8 (21.0)	11.6
SLP	Much improved	8	−29.6 (39.4)	−24.8	19	−36.8 (29.8)	−30.2	14	−23.8 (28.4)	−11.4
	Moderately improved	22	−24.2 (28.0)	−19.4	15	−18.9 (19.9)	−12.2	21	−20.6 (26.7)	−8.3
	Slightly improved	39	−10.9 (26.7)	−6.0	33	−15.5 (31.9)	−8.8	24	−16.9 (25.8)	−4.6
	No change	101	−4.8 (22.1)		67	−6.7 (25.7)		48	−12.4 (26.1)	
	Slightly worse	23	0.9 (19.0)	5.7	30	0.2 (18.8)	6.9	33	1.4 (22.0)	13.8
	Moderately worse	3	−5.6 (5.1)	−0.7	9	10.4 (17.0)	17.0	14	7.6 (32.6)	20.0
	Much worse				2	15.0 (21.2)	21.7	11	4.8 (21.0)	17.2
AUD	Much improved	8	−33.3 (29.2)	−36.3	19	-14.9 (22.1)	−11.5	14	−16.7 (25.1)	−14.2
	Moderately improved	22	−3.5 (18.4)	−6.4	15	−23.6 (26.7)	−20.2	21	−8.9 (28.0)	−6.4
	Slightly improved	39	−9.1 (18.2)	−12.1	33	1.5 (21.0)	4.9	24	7.5 (27.3)	10.0
	No change	101	2.9 (23.7)		67	−3.4 (18.8)		48	−2.5 (17.5)	
	Slightly worse	23	2.9 (26.8)	0.0	30	9.9 (24.2)	13.3	33	9.4 (25.5)	11.9
	Moderately worse	3	−3.3 (10.0)	−6.3	9	19.3 (22.3)	22.6	14	20.2 (32.7)	22.7
	Much worse				2	15.0 (21.2)	18.4	11	20.6 (22.2)	23.1
REL	Much improved	8	−52.9 (32.3)	−45.8	19	−38.9 (33.5)	−29.0	14	−42.4 (24.1)	−29.3
	Moderately improved	22	−23.6 (24.5)	−16.5	15	−21.1 (24.2)	−11.2	21	−25.2 (31.4)	−12.2
	Slightly improved	39	−16.2 (19.4)	−9.1	33	−17.5 (25.6)	−7.6	24	−17.5 (25.8)	-4.4
	No change	101	−7.1 (23.5)		67	−9.9 (21.7)		48	−13.1 (24.8)	
	Slightly worse	23	3.5 (23.6)	10.6	30	−2.3 (27.8)	7.6	33	−3.7 (22.6)	9.3
	Moderately worse	3	−5.6 (13.5)	1.5	9	−1.1 (8.7)	8.8	14	4.8 (34.8)	17.8
	Much worse				2	10.0 (14.1)	19.9	11	3.9 (10.9)	17.0
QOL	Much improved	8	−27.2 (27.6)	−23.7	19	−21.7 (19.6)	−15.6	14	−21.3 (20.9)	−16.9
	Moderately improved	22	−14.8 (22.8)	−11.3	15	−24.6 (24.4)	−18.5	21	−22.1 (27.1)	−17.7
	Slightly improved	39	−13.6 (14.3)	−10.1	33	−5.4 (17.8)	0.7	24	−9.3 (20.7)	−4.9
	No change	101	−3.5 (19.3)		67	−6.1 (20.6)		48	−4.4 (17.6)	
	Slightly worse	23	8.0 (21.7)	11.5	30	3.6 (23.5)	9.7	33	3.7 (20.9)	8.1
	Moderately worse	3	−10.0 (8.7)	−6.5	9	7.2 (28.2)	13.3	14	9.1 (24.2)	13.5
	Much worse				2	13.8 (19.4)	19.8	11	9.1 (19.0)	13.4
EMO	Much improved	8	−30.4 (20.8)	−25.4	19	−26.0 (24.1)	−20.7	14	−23.8 (27.2)	−14.1
	Moderately improved	22	−17.7 (23.8)	−12.7	15	−28.0 (26.6)	−22.7	21	−22.4 (24.2)	−12.7
	Slightly improved	39	−14.0 (20.8)	−9.0	33	−18.2 (24.6)	−12.9	24	−15.6 (21.6)	−5.8
	No change	101	−5.1 (17.6)		67	−5.3 (18.8)		48	−9.7 (22.5)	
	Slightly worse	23	0.9 (20.1)	5.9	30	−0.3 (23.9)	4.9	33	−6.6 (23.5)	3.2
	Moderately worse	3	7.8 (22.7)	12.8	9	0.0 (27.1)	5.3	14	4.0 (26.9)	13.8
	Much worse				2	6.7 (33.0)	11.9	11	7.6 (12.2)	17.3

INT = Intrusiveness, SOC = Sense of control, COG = Cognition, SLP = Sleep, AUD = Auditory, REL = Relaxation, QOL = Quality of life, and EMO = Emotional distress. T1 = 3-month follow-up, T2 = 6-month follow-up, T3 = 9-month follow-up; SD = standard deviation.

**Table 2:** Smallest Detectable Change (SDC) and Standard Error of Measurement (SEM) for Tinnitus Functional Index (TFI/TFI-22) scores between three administrations.

		Mean (±SD)			Difference		Measurement Error		
Scale	*n*	T0	T1	T2	Mean Diff	SD Diff	SEM	SEM × 4	SDC
TFI-25	50	50.8 (±25.1)	45.9 (±22.8)	44.9 (±23.1)	−5.4	7.2	5.1	20.4	14.2
TFI-22	50	51.3 (±25.9)	45.7 (±23.3)	45.0 (±23.7)	−5.9	7.1	5.0	20.0	13.9
INT	46	64.0 (±24.3)	55.6 (±23.2)	54.7 (±23.3)	−9.8	13.2	9.3	37.2	25.8
SOC	48	61.9 (±24.3)	56.5 (±24.2)	55.0 (±24.0)	−6.2	10.7	7.6	30.4	21.0
COG	49	40.9 (±28.4)	39.3 (±27.2)	38.0 (±26.0)	−2.2	13.5	9.6	38.4	26.5
SLP	48	52.6 (±32.0)	46.4 (±29.2)	47.1 (±29.4)	−4.8	13.3	9.4	37.6	25.9
AUD	50	47.7 (±30.1)	47.5 (±28.8)	44.4 (±28.2)	−1.7	16.2	10.7	42.8	29.6
REL	50	62.0 (±29.3)	55.9 (±26.4)	53.5 (±27.4)	−7.4	14.4	10.2	40.8	28.3
QOL	49	38.6 (±32.4)	34.7 (±28.9)	33.3 (±29.0)	−4.6	11.3	8.0	32.0	22.2
EMO	50	42.8 (±31.5)	35.7 (±29.7)	38.4 (±30.2)	−5.7	14.6	10.3	41.2	28.6

T0 = Baseline, T1 = 3-month follow-up, T2 = 6-month follow-up; Mean diff = the mean difference scores between administrations; SD diff = Standard Deviation of the difference; SEM = Standard Error in Measurement (SD*diff*/√2); SDC = Smallest Detectable Change (1.96 × √2 × SEM). For subscale definitions, please refer to Table 1.

**Table 3:** Mean (SD) TFI and TFI-22 global scores according to the Clinical Global Impression (CGI) categories stratified by TFI grading categories and the Minimal Clinically Important Difference (MCID) estimates for ‘improved’ at follow-up administrations.

	TFI Grading Categories	CGI-3	T1			T2			T3		
			*n*	Mean (SD)	MCID	*n*	Mean (SD)	MCID	*n*	Mean (SD)	MCID
TFI		Improved	8	−5.3 (9.6)		11	−5.7 (6.9)		11	−3.3 (8.1)	
	Small problem	No change	22	0.2 (9.1)	−5.5	13	−1.7 (7.1)	−4.0	10	−5.2 (8.9)	−4.0
		Worsened	3	9.2 (14.3)		5	5.4 (18.9)		8	12.7 (21.9)	
		Improved	20	−14.3 (10.3)		18	−17.3 (12.8)		15	−21.9 (12.0)	
	Moderate problem	No change	30	−2.3 (10.2)	−12.0	20	−1.5 (7.9)	−15.7	15	−5.4 (12.0)	−15.7
		Worsened	4	8.9 (14.1)		11	9.3 (12.5)		16	7.3 (17.0)	
		Improved	21	−16.7 (15.9)		23	−23.1 (16.1)		22	−20.4 (20.2)	
	Big problem	No change	22	−2.8 (11.8)	−13.9	13	−8.9 (10.2)	−14.2	9	−8.2 (15.0)	−14.2
		Worsened	10	−1.8 (9.8)		11	2.8 (14.6)		14	−1.0 (16.1)	
		Improved	20	−24.1 (19.6)		15	−26.6 (18.9)		11	−32.8 (22.8)	
	Very big problem	No change	27	−10.5 (13.8)	−13.6	21	−14.7 (18.4)	−11.9	14	−16.4 (12.1)	−11.9
		Worsened	9	0.5 (11.9)		14	−2.9 (14.2)		20	−1.8 (12.7)	
TFI-22		Improved	8	−5.3 (9.2)		11	−6.4 (6.9)		11	−5.7 (6.7)	
	Small problem	No change	22	0.2 (10.1)	−5.6	13	−1.8 (7.1)	−4.6	10	−4.6 (8.3)	−4.6
		Worsened	3	9.9 (14.9)		5	3.8 (22.2)		8	11.3 (22.4)	
		Improved	20	−15.6 (11.6)		18	−18.6 (14.0)		15	−24.3 (11.9)	
	Moderate problem	No change	30	−3.3 (10.2)	−12.3	20	−2.0 (9.3)	−16.6	15	−6.8 (12.7)	−16.6
		Worsened	4	10.6 (14.4)		11	7.9 (12.2)		16	5.8 (17.7)	
		Improved	21	−18.2 (17.6)		23	−25.6 (16.0)		22	−23.5 (20.6)	
	Big problem	No change	22	−4.3 (11.1)	−14.0	13	−9.4 (12.4)	−16.2	9	−9.2 (17.2)	−16.2
		Worsened	10	−2.7 (13.6)		11	2.3 (15.6)		14	−2.9 (18.6)	
		Improved	20	−24.8 (19.7)		15	−27.3 (18.6)		11	−33.8 (22.4)	
	Very big problem	No change	27	−11.2 (13.8)	−13.6	21	−15.5 (19.1)	−11.8	14	−17.8 (12.9)	−11.8
		Worsened	9	0.1 (12.6)		14	−4.1 (13.4)		20	−3.0 (12.5)	

T1 = 3-month follow-up, T2 = 6-month follow-up, T3 = 9-month follow-up; CGI-3: Clinical Global Impression collapsed to three categories: ‘improved’, ‘no change’, ‘worsened’.

**Table 4:** Characteristics of the Receiver Operating Characteristic (ROC) analysis and the optimum cut-off point for TFI-25, TFI-22 and subscales for detecting improvements and worsening of tinnitus impact across all administrations.

	T0–T1				T0–T2				T0–T3			
	Improved (69) vs. No Change (101)				Improved (67) vs. No Change (67)				Improved (59) vs. No Change (48)			
Scale	AUC (95%CI)	Optimal Value	Sens %	Spec %	AUC (95%CI)	Optimal Value	Sens %	Spec %	AUC (95%CI)	Optimal Value	Sens %	Spec %
TFI	0.75 (0.68–0.82)	−7.60	65	67	0.74 (0.66–0.83)	−10.2	68	67	0.70 (0.57–0.77)	−11.8	63	61
TFI-22	0.74 (0.67–0.82)	−7.50	68	66	0.75 (0.67–0.84)	−10.6	73	70	0.70 (0.59–0.79)	−12.0	61	57
INT	0.69 (0.60–0.77)	−11.66	63	69	0.68 (0.59–0.77)	−13.34	60	58	0.63 (0.52–0.74)	−18.34	59	56
SOC	0.69 (0.61–0.77)	−8.34	66	63	0.73 (0.64–0.82)	−13.34	67	66	0.64 (0.54–0.75)	−13.34	63	63
COG	0.61 (0.52–0.69)	−6.66	57	56	0.66 (0.56–0.75)	−8.34	60	61	0.65 (0.55–0.75)	−8.34	62	60
SLP	0.64 (0.56–0.73)	−8.34	57	57	0.67 (0.57–0.76)	−11.66	64	67	0.63 (0.53–0.74)	−16.65	64	65
AUD	0.64 (0.56–0.73)	−1.66	53	59	0.56 (0.46–066)	−1.66	54	54	0.54 (0.43–0.65)	−1.66	54	56
REL	0.69 (0.60–0.77)	−13.33	61	69	0.64 (0.55–0.74)	−16.67	61	61	0.64 (0.54–0.75)	−15	61	60
QOL	0.69 (0.61–0.77)	−6.25	65	65	0.62 (0.53–0.72)	−6.25	60	58	0.66 (0.56–0.77)	−6.25	61	63
EMO	0.66 (0.57–0.74)	−8.34	62	68	0.73 (0.64–0.82)	−8.34	65	70	0.63 (0.52–0.73)	−11.67	55	65
	**Worsened (26) vs. No change (101)**				**Worsened (41) vs. No change (67)**				**Worsened (58) vs. No change (48)**			
Scale	AUC (95% CI)	Optimal value	Sens %	Spec %	AUC (95% CI)	Optimal value	Sens %	Spec %	AUC (95%CI)	Optimal value	Sens %	Spec %
TFI	0.63 (0.50–0.75)	1.4	62	58	0.66 (0.56–0.77)	4.0	61	59	0.70 (0.61–0.80)	2.8	64	62
TFI-22	0.64 (0.51–0.78)	1.6	59	58	0.64 (0.53–0.75)	3.9	56	56	0.70 (0.60–0.80)	4.1	64	64

T0 = Baseline, T1 = 3-month follow-up, T2 = 6-month follow-up, T3 = 9-month follow-up; AUC = Area Under the Curve; Sens = sensitivity; Spec = specificity. For subscale definitions, please refer to Table 1.

## Data Availability

The datasets used and/or analysed during the current study are available from the corresponding author on reasonable request.

## Supplementary Materials

The following supporting information can be downloaded at: https://www.mdpi.com/article/10.3390/brainsci12060726/s1, Figure S1: Receiver Operating Characteristic (ROC) curves with optimal values for identifying ‘improvements’ above ‘no change’ using the TFI-22 global change scores; Figure S2: Distributions (expressed in percent) of the changes in the TFI-22 global scores for tinnitus patients who reported improvements in tinnitus (blue distribution line) and those who reported no change in tinnitus at 3 months from baseline (red distribution line); Table S1: Descriptive statistics for the TFI, TFI-22 and subscales; Table S2: Mean (SD) TFI, TFI-22 global and subscale scores according to three Clinical Global Impression (CGI) categories and Minimal Clinically Important Difference (MCID) for ‘improved’ and ‘worsened’ categories for each subscale at follow-up administrations; Table S3: Spearman’s Rho correlations between the TFI, TFI-22 global and subscale score and the Clinical Global Impression (CGI) categories.

## References

[1] Watts E.J., Fackrell K., Smith S., Sheldrake J., Haider H., Hoare D.J. (2018). Why Is Tinnitus a Problem? A Qualitative Analysis of Problems Reported by Tinnitus Patients. Trends Hear..

[2] Baguley D., McFerran D., Hall D. (2013). Tinnitus. Lancet.

[3] Hall D.A., Haider H., Szczepek A.J., Lau P., Rabau S., Jones-Diette J., Londero A., Edvall N.K., Cederroth C.R., Mielczarek M. (2016). Systematic review of outcome domains and instruments used in clinical trials of tinnitus treatments in adults. Trials.

[4] Fackrell K., Hall D.A., Barry J.G., Hoare D.J., Signorelli F., Turjman F. (2014). Tools for tinnitus measurement: Development and validity of questionnaires to assess handicap and treatment effects. Tinnitus: Causes, Treatment and Short & Long-Term Health Effects.

[5] Meikle M., Henry J., Griest S., Stewart B., Abrams H., McArdle R., Myers P., Newman C., Sandridge S., Turk D. (2012). The Tinnitus Functional Index: Development of a new clinical measure for chronic, intrusive tinnitus. Ear Hear..

[6] Henry J.A., Griest S., Thielman E., McMillan G., Kaelin C., Carlson K.F. (2016). Tinnitus Functional Index: Development, validation, outcomes research, and clinical application. Hear. Res..

[7] Skarżyński H., Gos E., Dziendziel B., Raj-Koziak D., Włodarczyk E.A., Skarżyński P.H. (2018). Clinically important change in tinnitus sensation after stapedotomy. Health Qual. Life Outcomes.

[8] Fackrell K., Hall D.A., Barry J.G., Hoare D.J. (2016). Psychometric properties of the Tinnitus Functional Index (TFI): Assessment in a UK research volunteer population. Hear. Res..

[9] Folmer R.L. (2016). Reply to: Psychometric properties of the Tinnitus Functional Index (TFI): Assessment in a UK research volunteer population. Hear. Res..

[10] Folmer R.L., Theodoroff S.M., Casiana L., Shi Y., Griest S., Vachhani J. (2015). Repetitive transcranial magnetic stimulation treatment for chronic tinnitus: A randomized clinical trial. JAMA Otolaryngol.-Head Neck Surg..

[11] Chandra N., Chang K., Lee A., Shekhawat G.S., Searchfield G.D. (2018). Psychometric validity, reliability, and responsiveness of the tinnitus functional index. J. Am. Acad. Audiol..

[12] Fackrell K., Hall D.A., Barry J.G., Hoare D.J. (2018). Performance of the Tinnitus Functional Index as a diagnostic instrument in an UK clinical population. Hear. Res..

[13] Crosby R.D., Kolotkin R.L., Williams G.R. (2004). An integrated method to determine meaningful changes in health-related quality of life. J. Clin. Epidemiol..

[14] Yost K.J., Eton D.T. (2005). Combining distribution- and anchor-based approaches to determine minimally important differences: The FACIT experience. Eval. Health Prof..

[15] De Vet H.C.W., Ostelo R.W.J.G., Terwee C.B., Van Der Roer N., Knol D.L., Beckerman H., Boers M., Bouter L.M. (2007). Minimally important change determined by a visual method integrating an anchor-based and a distribution-based approach. Qual. Life Res..

[16] Vernon J., Griest S., Press L. (1992). Plight of unreturned tinnitus questionnaires. Br. J. Audiol..

[17] IBM Corp. (2012). IBM SPSS Statistics for Window, Version 21.0.

[18] Lipsey M.W. (1983). A scheme for assessing measurement sensitivity in program evaluation and other applied research. Psychol. Bull..

[19] Crosby R.D., Kolotkin R.L., Williams G.R. (2003). Defining clinically meaningful change in health-related quality of life. J. Clin. Epidemiol..

[20] Revicki D., Hays R.D., Cella D., Sloan J. (2008). Recommended methods for determining responsiveness and minimally important differences for patient-reported outcomes. J. Clin. Epidemiol..

[21] Terwee C.B., Roorda L.D., Knol D.L., De Boer M.R., De Vet H.C.W.W., De Boer M.R., Vet H.C.W. (2009). De Linking measurement error to minimal important change of patient-reported outcomes. J. Clin. Epidemiol..

[22] De Vet H.C.W., Terwee C.B., Mokkink L.B., Knol D.L. (2011). Measurement in Medicine: A Practical Guide.

[23] Copay A.G., Subach B.R., Glassman S.D., Polly D.W., Schuler T.C. (2007). Understanding the minimum clinically important difference: A review of concepts and methods. Spine J..

[24] Rai S.K., Yazdany J., Fortin P.R., Antonio Aviña-Zubieta J. (2015). Approaches for estimating minimal clinically important differences in systemic lupus erythematosus. Arthritis Res. Ther..

[25] Bland J.M., Altman D.G. (2007). Agreement between methods of measurement with multple observations per individual. J. Biopharm. Stat..

[26] Weir J.P. (2005). Quantifying test-retest reliability using the intraclass correlation coefficient and the SEM. J. Strength Cond. Res..

[27] Streiner D.L., Norman G.R. (2008). Health Measurement Scales: A practical Guide to Their Development and Use.

[28] Wyrwich K.W., Norquist J.M., Lenderking W.R., Acaster S. (2013). Methods for interpreting change over time in patient-reported outcome measures. Qual. Life Res..

[29] Terwee C.B., Bot S.D.M., de Boer M.R., van der Windt D., Knol D.L., Dekker J., Bouter L.M., de Vet H.C.W. (2007). Quality criteria were proposed for measurement properties of health status questionnaires. J. Clin. Epidemiol..

[30] Cohen J. (1988). Statistical Power Analysis for the Behavioral Sciences.

[31] Cohen J. (1992). A power primer. Psychol. Bull..

[32] Norman G.R., Sloan J.A., Wyrwich K.W. (2003). Interpretation of changes in health-related quality of life: The remarkable universality of half a standard deviation. Med. Care.

[33] Angst F., Aeschlimann A.E., Angst J. (2017). The minimal clinically important difference raised the significance of outcome effects above the statistical level, with methodological implications for future studies. J. Clin. Epidemiol..

[34] De Vet H.C.W., Terluin B., Knol D.L., Roorda L.D., Mokkink L.B., Ostelo R.W.J.G., Hendriks E.J.M., Bouter L.M., Terwee C.B. (2010). Three ways to quantify uncertainty in individually applied “minimally important change” values. J. Clin. Epidemiol..

[35] Terwee C.B., Roorda L.D., Dekker J., Bierma-Zeinstra S.M., Peat G., Jordan K.P., Croft P., de Vet H.C.W. (2010). Mind the MIC: Large variation among populations and methods. J. Clin. Epidemiol..

[36] Ward M.M., Guthrie L.C., Alba M. (2014). Dependence of the minimal clinically important improvement on the baseline value is a consequence of floor and ceiling effects and not different expectations by patients. J. Clin. Epidemiol..

[37] De Vet H.C.W., Foumani M., Scholten M.A., Jacobs W.C.H., Stiggelbout A.M., Knol D.L., Peul W.C. (2015). Minimally important change values of a measurement instrument depend more on baseline values than on the type of intervention. J. Clin. Epidemiol..

[38] Eng J. (2005). Receiver operating characteristic analysis: A primer. Acad. Radiol..

[39] Uslu R.I., Kapci E.G., Oncu B., Ugurlu M., Turkcapar H. (2008). Psychometric properties and cut-off scores of the beck depression inventory-II in Turkish adolescents. J. Clin. Psychol. Med. Settings.

[40] Zou K.H., Malley A.J.O., Mauri L., O’Malley A.J., Mauri L. (2007). Receiver-operating characteristic analysis for evaluating diagnostic tests and predictive models. Circulation.

[41] Henry J.A., Thielman E., Zaugg T. (2017). Reply to: Psychometric properties of the Tinnitus Functional Index (TFI): Assessment in a UK research volunteer population. Hear. Res..

[42] Fackrell K., Hall D.A., Barry J.G., Hoare D.J. (2016). Response to Letter: Psychometric properties of the Tinnitus Functional Index (TFI): Assessment in a UK research volunteer population. Hear. Res..

[43] Fackrell K., Hall D.A., Barry J.G., Hoare D.J. (2017). Response to letter: Psychometric properties of the Tinnitus Functional Index (TFI): Assessment in a UK research volunteer population. Hear. Res..

[44] Sirois F.M., Davis C.G., Morgan M.S. (2006). “Learning to live with what you can’t rise above”: Control beliefs, symptom control, and adjustment to tinnitus. Health Psychol..

[45] Vollmann M., Kalkouskaya N., Langguth B., Scharloo M. (2012). When the ringing in the ears gets unbearable: Illness representations, self-instructions and adjustment to tinnitus. J. Psychosom. Res..

[46] Dawes P., Newall J., Stockdale D., Baguley D.M. (2020). Natural history of tinnitus in adults: A cross-sectional and longitudinal analysis. BMJ Open.

[47] Searchfield G.D. (2014). Tinnitus what and where: An ecological framework. Front. Neurol..

[48] Mokkink L.B., Terwee C.B., Patrick D.L., Alonso J., Stratford P.W., Knol D.L., Bouter L.M., de Vet H.C.W. (2012). The COSMIN Checklist Manual.

[49] Lipsey M.W., Cordray D. (2000). Evaluation methods for social intervention. Annu. Rev. Psychol..

[50] Adamchic I., Tass P.A., Langguth B., Hauptmann C., Koller M., Schecklmann M., Zeman F., Landgrebe M. (2012). Linking the Tinnitus Questionnaire and the subjective Clinical Global Impression: Which differences are clinically important?. Health Qual. Life Outcomes.

[51] Phillips J.S., McFerran D.J., Hall D.A., Hoare D.J. (2018). The natural history of subjective tinnitus in adults: A systematic review and meta-analysis of no-intervention periods in controlled trials. Laryngoscope.

