# Supplementary material for: Integrating Distribution-Based and Anchor-Based Techniques to Identify Minimal Important Change for the Tinnitus Functional Index (TFI) Questionnaire
Source: Brain Sci. 2022 May 31;12(6):726. doi: 10.3390/brainsci12060726 (PMC9220811; doi:10.3390/brainsci12060726)
Supplement: Supplementary file 1 [file brainsci-12-00726-s001.zip › brainsci-1717173 - supp - proofread_KFackrell.pdf]

Table S1. Descriptive statistics for the TFI, TFI-22 and subscales.

			**T0**		**T1**		**T2**		**T3**	
	**Scale**	**Range**	***n***	**Mean (SD)**	***n***	**Mean (SD)**	***n***	**Mean (SD)**	***n***	**Mean (SD)**
	TFI		255	52.7 (21.7)	196	44.7 (22.4)	175	43.0 (23.7)	165	42.9 (25.5)
Grading	No problem	0–7	–	–	–	–	–	–	–	–
	Small problem	7–28	38	20.6 (6.2)	32	21.2 (10.1)	28	19.1 (10.0)	28	21.8 (15.8)
	Moderate problem	29–47	72	38.5 (50.2)	53	33.3 (13.4)	49	33.7 (15.8)	46	32.0 (19.0)
	Big problem	48–65	70	56.5 (5.5)	53	47.2 (15.3)	46	42.9 (18.6)	44	43.6 (20.8)
	Very big problem	66–100	75	79.1 (10.0)	58	65.9 (20.0)	52	64.6 (21.3)	47	65.6 (22.0)
	TFI22		255	54.1 (22.4)	195	54,1 (23.2)	175	43.3 (24.3)	164	42.8 (25.9)
Grading	No problem	0–7	–	–	–	–	–	–	–	–
	Small problem	7–28	31	18.4 (5.4)	26	19.9 (10.6)	24	17.7 (11.2)	24	19.2 (14.6)
	Moderate problem	29–47	80	38.9 (6.4)	59	32.1 (13.5)	53	32.3 (15.8)	50	30.6 (18.6)
	Big problem	48–65	81	60.0 (6.7)	63	49.7 (15.6)	57	46.9 (18.8)	54	45.2 (19.5)
	Very big problem	66–100	63	83.4 (8.5)	48	69.2 (20.7)	41	67.3 (22.6)	37	70.9 (21.6)
Subscales	INTR		251	62.3 (22.0)	191	52.3 (23.8)	163	50.7 (25.2)	157	48.1 (25.8)
	SOC		251	64.5 (21.7)	196	54.4 (24.6)	173	51.0 (25.7)	164	52.1 (27.4)
	COG		255	47.1 (26.7)	193	41.0 (26.1)	175	39.3 (27.1)	165	38.2 (28.3)
	SLP		253	55.6 (31.9)	196	45.2 (30.6)	175	42.4 (31.1)	164	40.8 (33.2)
	AUD		254	42.6 (30.7)	194	40.7 (28.4)	175	40.7 (28.7)	165	44.2 (30.6)
	REL		254	64.4 (27.8)	195	53.6 (26.7)	173	51.4 (28.3)	163	50.9 (29.4)
	QOL		255	39.9 (29.5)	196	33.7 (27.3)	175	33.8 (27.8)	165	34.2 (29.0)
	EMO		255	49.4 (30.4)	195	39.9 (29.6)	175	37.7 (30.0)	165	37.3 (30.9)

The maximum score is 100. SD = Standard Deviation; T0 = baseline; T1 = 3-month follow-up, T2 = 6-month follow-up, T3 = 9-month follow-up. – = no data

Table S2. Mean (SD) TFI, TFI-22 global and subscale scores according to three Clinical Global Impression (CGI) categories and Minimal Clinically Important Difference (MCID) for 'improved' and 'worsened' categories for each subscale at follow-up administrations.

		**T1**			**T2**			T3		
	**CGI-3**	***n***	**Mean (SD)**	**MCID**	***n***	**Mean (SD)**	**MCID**	***n***	**Mean (SD)**	**MCID**
TFI-25	Improved	69	—16.8 (15.9)	−12.7	67	−19.4 (16.1)	−12.3	59	−19.9 (19.2)	−10.8
	No change	101	−4.1 (12.0)		67	−7.1 (13.5)		48	−9.1 (12.7)	
	Worsened	26	1.9 (11.8)	6	41	2.9 (14.7)	10	58	2.9 (16.7)	12
TFI–22	Improved	69	−17.9 (16.8)	−13.0	67	−20.9 (16.4)	−13.3	59	−22.32 (19.0)	−12.3
	No change	101	−4.9 (12.0)		67	−7.6 (14.5)		48	−10.0 (13.6)	
	Worsened	26	1.7 (13.7)	6.6	41	1.8 (15.1)	9.4	58	1.4 (17.5)	11.4
INT	Improved	69	−20.1 (21.7)	−13.1	67	−25.7 (22.4)	−13.8	59	−27.9 (20.4)	−9.7
	No change	101	−7.0 (22.4)		67	−11.9 (18.1)		48	−18.2 (22.6)	
	Worsened	26	−5.0 (22.8)	2.0	41	3.7 (21.3)	15.5	58	−2.5 (23.2)	15.7
SOC	Improved	69	−20.7 (25.1)	−16.6	67	−25.9 (22.2)	−16.1	59	−23.1 (25.5)	−13.0
	No change	101	−4.2 (18.3)		67	−9.8 (23.7)		48	−10.1 (20.2)	
	Worsened	26	3.7 (22.8)	7.9	41	0.7 (23.2)	10.5	58	−0.6 (22.0)	9.6
COG	Improved	69	−12.2 (21.8)	−7.4	67	−15.5 (20.4)	−10.7	59	−19.9 (26.4)	−13.2
	No change	101	−4.8 (21.6)		67	−4.8 (19.5)		48	−6.7 (15.4)	
	Worsened	26	2.8 (20.2)	7.6	41	2.8 (20.0)	7.7	58	3.6 (24.5)	10.3
SLP	Improved	69	−17.3 (29.3)	−12.5	67	−22.3 (30.1)	−15.6	59	−24.7 (28.8)	−12.4

	No change	101	−4.8 (22.1)		67	−6.7 (25.7)		48	−12.4 (26.1)	
	Worsened	26	0.1 (18.0)	4.9	41	3.2 (18.7)	9.8	58	2.5 (24.6)	14.9
AUD	Improved	69	−10.1 (21.3)	−13.1	67	−8.8 (24.7)	−5.4	59	−4.1 (28.5)	−1.6
	No change	101	2.9 (23.7)		67	−3.4 (18.8)		48	−2.5 (17.5)	
	Worsened	26	2.2 (25.4)	−0.8	41	12.2 (23.4)	15.6	58	14.1 (26.9)	16.6
REL	Improved	69	−22.8 (25.2)	−15.7	67	−24.4 (28.9)	−14.5	59	−26.2 (31.0)	−13.1
	No change	101	−7.1 (23.5)		67	−9.9 (21.7)		48	−13.1 (24.8)	
	Worsened	26	2.4 (22.7)	9.5	41	−1.5 (24.2)	8.4	58	−0.2 (24.5)	12.8
QOL	Improved	69	−15.5 (19.3)	−12.0	67	−14.3 (21.6)	−8.2	59	−16.7 (23.7)	−12.3
	No change	101	−3.5 (19.3)		67	−6.1 (20.6)		48	−4.4 (17.6)	
	Worsened	26	6.0 (21.3)	9.5	41	4.9 (24.0)	11.0	58	6.0 (21.2)	10.4
EMO	Improved	69	−17.1 (22.1)	−12.1	67	−22.6 (24.9)	−17.3	59	−19.9 (23.9)	−10.2
	No change	101	−5.1 (17.6)		67	−5.3 (18.8)		48	−9.7 (22.5)	
	Worsened	26	1.7 (20.1)	6.7	41	0.1 (24.3)	5.4	58	−1.3 (23.2)	8.4

T1 = 3mth follow-up, T2 = 6mth follow-up, T3 = 9mth follow-up; CGI-3: Clinical Global Impression collapsed to three categories: ‘improved’, ‘no change’, ‘worsened’; SD = Standard deviation.

Table S3. Spearman’s Rho correlations between the TFI, TFI−22 global and subscale score and the Clinical Global Impression (CGI) categories.

		T0-T1		T0-T2		T0-T3	
		CGI-7	CGI-3	CGI-7	CGI-3	CGI-7	CGI-3
Global	TFI	**−0.44**	**−0.45**	**−0.54**	**−0.51**	**−0.53**	**−0.48**
	TFI-22	**−0.41**	**−0.45**	**−0.53**	**−0.50**	**−0.54**	**−0.50**
Subscales	INT	−0.30	−0.33	**−0.50**	**−0.48**	**−0.54**	**−0.49**
	SOC	−0.33	−0.36	**−0.49**	**−0.47**	**−0.43**	−0.39
	COG	−0.21	−0.22	−0.34	−0.32	−0.38	−0.37
	SLP	−0.27	−0.29	**−0.42**	−0.39	**−0.46**	**−0.42**
	AUD	−0.18	−0.22	−0.38	−0.33	−0.37	−0.31
	REL	−0.31	−0.33	−0.36	−0.34	**−0.40**	−0.38
	QOL	−0.29	−0.33	−0.33	−0.29	**−0.44**	**−0.40**
	EMO	−0.27	−0.30	**−0.40**	−0.38	−0.35	−0.31

CGI-7: Clinical Global Impression categories: ‘much improved’, ‘moderately improved’, ‘slightly improved’, ‘no change’, ‘slightly worse’, ‘moderately worse’, ‘much worse’; CGI-3: Clinical Global Impression collapsed to three categories: ‘improved’, ‘no change’, ‘worsened’. Values presented in bold indicate correlations that meet the recommended criteria (Spearman’s Rho <0.4).

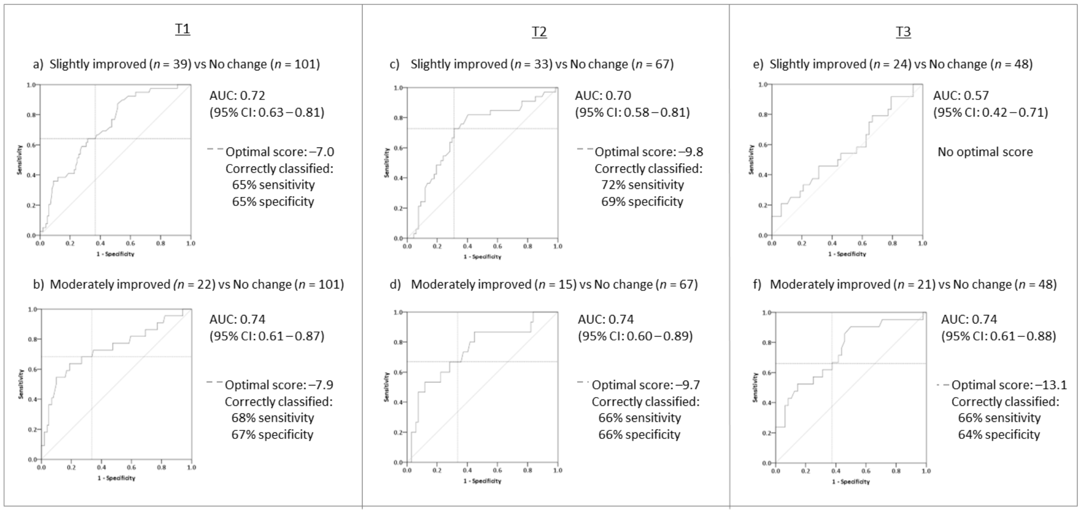

Figure S1. Receiver Operating Characteristic (ROC) curves with optimal values for identifying 'improvements' above 'no change' using the TFI-22 global change scores. a,c,e) = patients who reported 'slight improvements' with those who reported 'no change' in their tinnitus for T1 (3 mths), T2 (6 mths), and T3 (9 mths); (b,d,f) = patients who reported 'moderate improvements' with those who reported 'no change' in their tinnitus for T1 (3 mths), T2 (6 mths), and T3 (9 mths); solid light grey line indicates 50% probability of correctly classifying im-provement. AUC = Area Under the Curve.

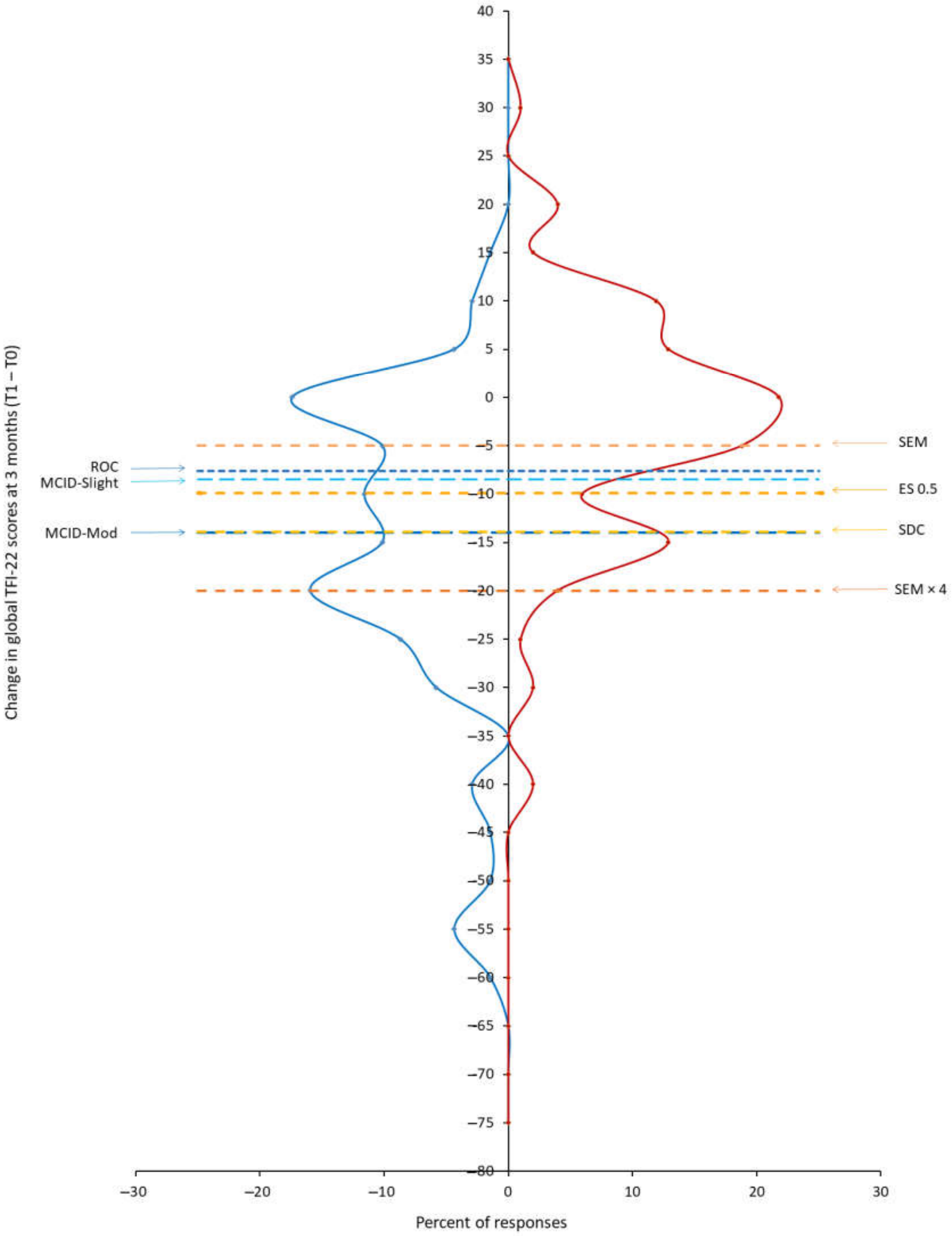

Figure S2. Distributions (expressed in percent) of the changes in the TFI-22 global scores for tinnitus patients who reported improvements in tinnitus (blue distribution line) and those who reported no change in tinnitus at 3 months from baseline (red distribution line).
