# Supplementary figures and images for: Integrating Distribution-Based and Anchor-Based Techniques to Identify Minimal Important Change for the Tinnitus Functional Index (TFI) Questionnaire
Source: Brain Sci. 2022 May 31;12(6):726. doi: 10.3390/brainsci12060726 (PMC9220811; doi:10.3390/brainsci12060726)

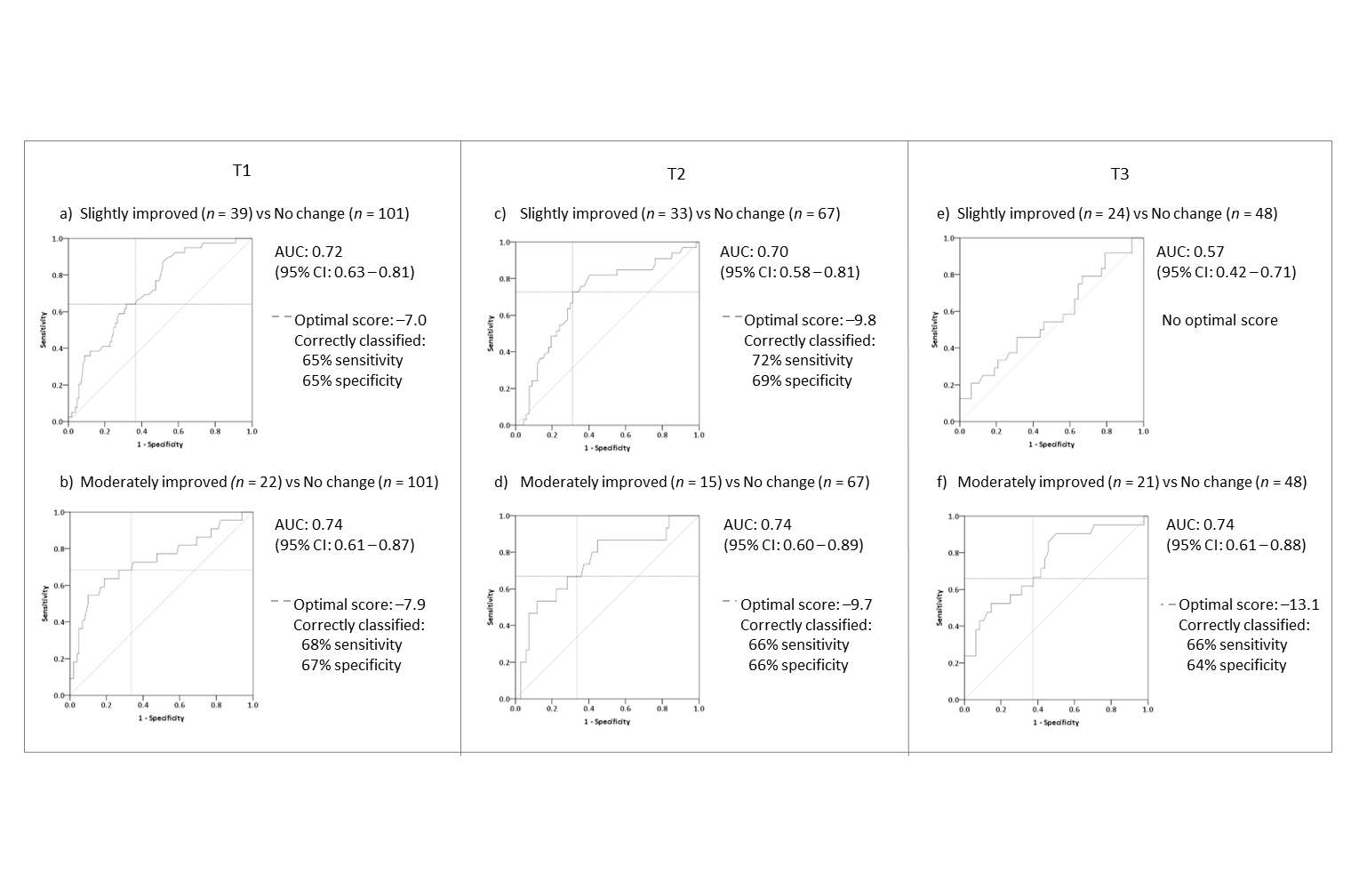

Supplement: Supplementary file 1 [file brainsci-12-00726-s001.zip › Supplementary Figure S1 - ROC TFI-22.tif]

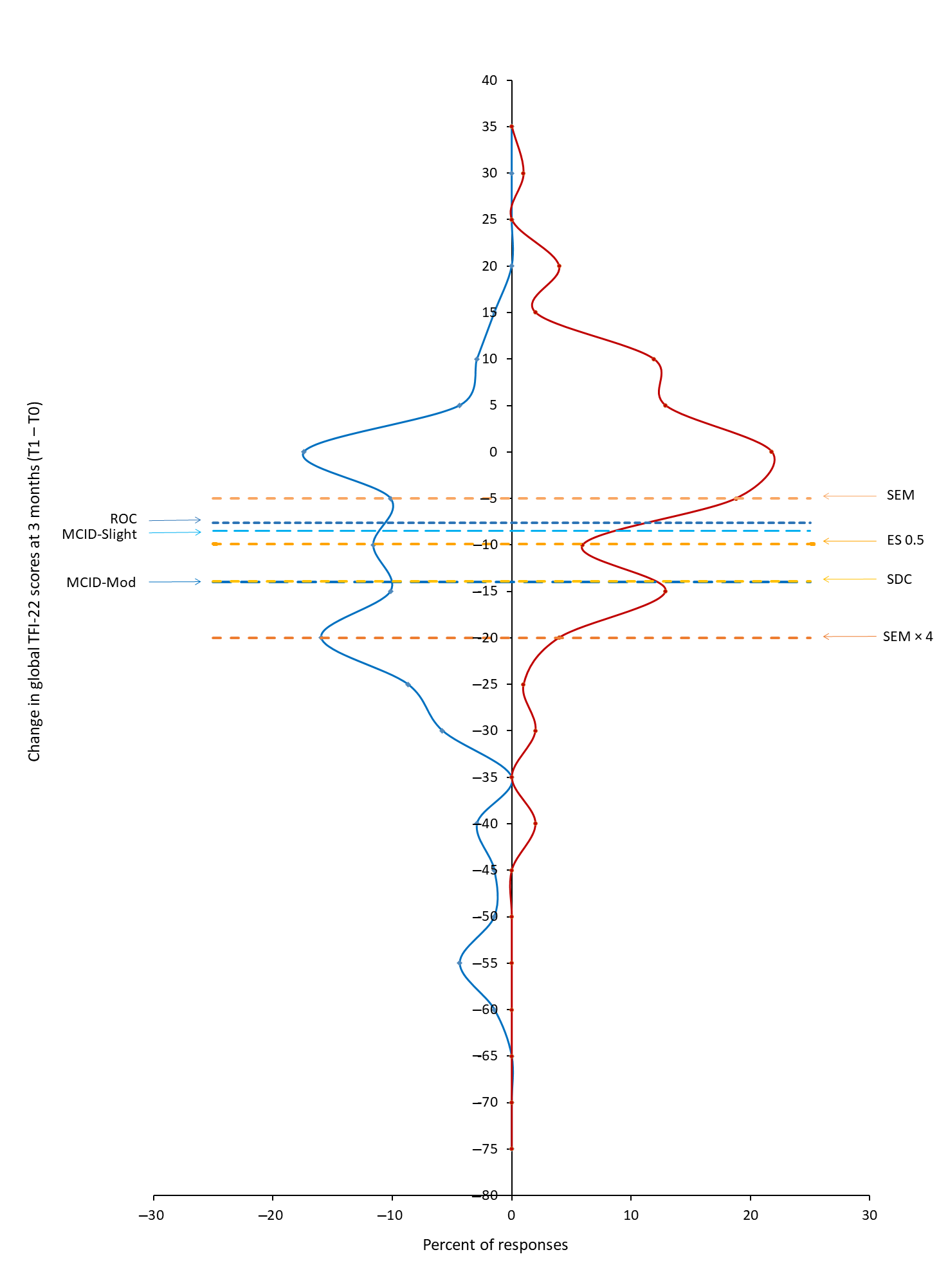

Supplement: Supplementary file 1 [file brainsci-12-00726-s001.zip › Supplementary Figure S2 - Visual plot TFI-22.tif]
